# Nectin-4 as a Predictive Marker for Poor Prognosis of Endometrial Cancer with Mismatch Repair Impairment

**DOI:** 10.3390/cancers15102865

**Published:** 2023-05-22

**Authors:** Ha Kyun Chang, Young Hoon Park, Jung-A Choi, Jeong Won Kim, Jisup Kim, Hyo Sun Kim, Hae Nam Lee, Hanbyoul Cho, Joon-Yong Chung, Jae-Hoon Kim

**Affiliations:** 1 Department of Obstetrics and Gynecology, Korea University Ansan Hospital, Korea University School of Medicine, Ansan 15355, Republic of Korea; coolblue23@naver.com; 2 Obstetrics and Gynecology, Yonsei University College of Medicine, Seoul 03722, Republic of Korea; skyblue3691@yuhs.ac (Y.H.P.); khs88@yuhs.ac (H.S.K.); hanbyoul@yuhs.ac (H.C.); 3 Department of Obstetrics and Gynecology, Gangnam Severance Hospital, Yonsei University College of Medicine, Seoul 06229, Republic of Korea; 4 Institute of Women’s Life Medical Science, Yonsei University College of Medicine, Seoul 03722, Republic of Korea; 5 Department of Pathology, Kangnam Sacred Heart Hospital, Hallym University College of Medicine, Seoul 07441, Republic of Korea; jwkim@hallym.or.kr; 6 Department of Pathology, Gil Medical Center, Gachon University College of Medicine, Incheon 21565, Republic of Korea; jspath@gilhospital.com; 7 Department of Obstetrics and Gynecology, Catholic University of Korea Bucheon St. Mary’s Hospital, Bucheon 14647, Republic of Korea; leehn@catholic.ac.kr; 8 Molecular Imaging Branch, National Cancer Institute, Center for Cancer Research, National Institutes of Health, Bethesda, MD 20892, USA; chungjo@mail.nih.gov

**Keywords:** Nectin-4, endometrial cancer, MSH2, MSH6

## Abstract

**Simple Summary:**

Endometrial cancer has become increasingly common owing to the recent westernization of diet and lifestyle, with 1.7% of cancer patients dying annually. Furthermore, the 5-year endometrial cancer survival rate is barely 15%. Nectin-4 has emerged as a possible biomarker and therapeutic target. Nectin-4 is highly expressed in various cancers. However, no studies have been conducted to determine the clinical importance of Nectin-4 expression in endometrial cancer. Here, we examined 320 tissue samples from patients with endometrial cancer to determine the relevance of Nectin-4 expression in the diagnosis and prognosis of endometrial cancer. Our findings emphasize the importance of Nectin-4 as a novel diagnostic tool and screening marker for assessing endometrial cancer and improving the accuracy of approaches used to predict high-risk endometrial cancer.

**Abstract:**

The adhesion molecule Nectin-4 is a new potential therapeutic target for different types of cancer; however, little is known about its diagnosis significance in endometrial cancer (EC). We found that Nectin-4 expression was significantly higher in EC tissues than in nonadjacent normal tissue. The area under the receiver operating characteristic curve value of 0.922 indicated good diagnostic accuracy for Nectin-4 expression in EC. Furthermore, Nectin-4 expression was associated with DNA mismatch repair (MMR) protein deficiency. Notably, the high Nectin-4 expression group of patients with MSH2/6-deficient EC had shorter progression-free survival than that of the low Nectin-4 expression group. The number of lymphovascular space invasion-positive patients in groups with MMR deficiency and high Nectin-4 expression was also increased compared with that in the low Nectin-4 expression group. Bioinformatics analysis revealed that alteration in Nectin-4 and MMR genes is associated with Nectin-4 expression in EC. To the best of our knowledge, this is the first study to show that Nectin-4 expression may be a potential biomarker for EC diagnosis and that high Nectin-4 expression in MMR-deficient patients with EC can predict short progression-free survival, thus providing clues to identify patients for adjuvant therapy.

## 1. Introduction

Endometrial cancer (EC) is the seventh most common cancer in women. EC is becoming increasingly prevalent because of the recent westernization of diet and lifestyle, with 1.7% of women with cancer dying of EC annually [1]. Although most women show signs following the onset of postmenopausal bleeding and have a good prognosis, 20% of women have advanced disease, and the 5-year survival rate is only 15% [2]. Therefore, new strategies are required to identify biomarkers for early EC detection and high-risk monitoring.

The mismatch repair (MMR) system plays a critical role in DNA replication by recognizing and fixing incorrectly paired nucleotides [3]. Thus, disruption of DNA MMR, which safeguards DNA integrity, is associated with an increased risk of developing several types of cancer including colon and ECs [3,4]. MMR deficiency is a frequent event in EC, with reported rates ranging from 20 to 40% [5]. MMR deficiency results from somatic or germline (inherited) mutations and is most commonly observed in *MLH1*, *MSH2*, *MSH6*, and *PMS2* genes [6]. MLH1, PMS2, MSH2, and MSH6 proteins are mainly detected using immunohistochemical methods [7]. Although MMR deficiency has been extensively studied as a predictive and prognostic biomarker of EC, the impact of MMR status on prognosis remains unclear. MMR deficiency predicts the presence of high-risk features of the disease, including advanced stages and uterine risk factors [8,9,10,11,12,13,14]. Aberrant *MSH2* expression is associated with aggressive prostate cancer and more rapid progression to biochemical recurrence [8]. However, a meta-analysis including all histologic subtypes suggested no impact of MMR status on the prognoses of ECs [15]. No differences in risk associations by MMR status have been observed for menopausal hormone use, parity, and age at menarche [16]. The consistent association between MMR and poor outcomes remains unknown. Therefore, the association between aberrant *MSH2* expression and aggressive EC is worth noting.

Nectin-4 (PVRL4—poliovirus receptor-related protein 4) is a Ca^2+^-independent immunoglobulin-like cell adhesion molecule that has been recently identified as an epithelial cell receptor for the measles virus [17,18]. Nectin-4 belongs to the Nectin family, which comprises four members (Nectin-1, -2, -3, and -4) and mediates various cell functions, such as proliferation, differentiation, migration, and invasion [19]. Recently, Nectin-4 has emerged as a potential biomarker and a promising target for therapy. Nectin-4 is particularly overexpressed in various cancers, including bladder [20], lung [21], ovarian [22], pancreatic [23], and gastric cancers [24]. Nectin-4 has a critical role in cancer cell proliferation and metastasis [23,25,26,27] via the proliferation of cancer cells by activating the phosphatidylinositol-3 kinase (PI3K)/Akt pathway [27,28] and epithelial–mesenchymal transition (EMT)-related signaling, which contributes to the metastasis of tumor cells [29]. Importantly, enfortumab vedotin, an antibody–drug conjugate (ADC) that targets Nectin-4, has been evaluated as a monotherapy in combination with a checkpoint inhibitor or chemotherapy in locally advanced and metastatic urothelial carcinoma [30]. However, the clinical significance of Nectin-4 as a biomarker or targeted therapy according to Nectin-4 expression in EC has not been reported.

Here, we assessed the diagnostic value of Nectin-4 expression for the diagnosis and prognosis of EC. In addition, we highlighted the clinical significance of the Nectin-4 expression pattern in MSH-deficient ECs.

## 2. Materials and Methods

### 2.1. Tissue Specimens

In total, 320 EC, 54 endometrial intraepithelial neoplasia (EIN), and 87 nonadjacent normal tissue samples were obtained from patients at Gangnam Severance Hospital, Seoul, South Korea. The tissue microarray paraffin blocks were provided by the Korea Gynecologic Cancer Bank. Tissue samples were collected from patients after they provided written informed consent. Table S1 shows the inclusion and exclusion criteria for the retrospective study. EC stages were assigned according to the International Federation of Gynecology and Obstetrics (FIGO) classification, and tumors were graded according to the World Health Organization grading system. Clinical and pathological records were reviewed to collect data, including age, surgical procedure, survival time, survival status, tumor grade, and cell type.

### 2.2. Tissue Microarray Construction and Immunohistochemistry

Tissue microarrays were produced from archival formalin-fixed, paraffin-embedded tissue blocks, and representative areas were meticulously selected from hematoxylin and eosin-stained slides. Tissue cylinders (1.0 mm diameter) were extracted from selected areas of donor blocks and transplanted into recipient blocks using a tissue arrayer (Beecher Instruments, Inc., Silver Spring, MD, USA). For immunohistochemical staining, the tissue microarray blocks were cut into serial 5 mm thick sections, deparaffinized in xylene, and rehydrated through a graded alcohol series to distilled water. Heat-induced antigen retrieval was performed for 20 min in an antigen retrieval buffer at pH 6 (Dako, Carpinteria, CA, USA). The endogenous peroxidase activity was blocked by incubation with 3% H_2_O_2_ for 10 min. Next, the sections were incubated with an anti-Nectin-4 mouse monoclonal antibody (clone no. G-2; Santa Cruz Biotechnology, Dallas, TX, USA) at a 1:200 dilution for 1 h. The antigen–antibody reaction was detected using the EnVision+ Dual Link System-HRP (Dako) and visualized with 3,3′-diaminobenzidine (Dako). Sections were lightly counterstained with hematoxylin. Appropriate negative and positive controls were analyzed concurrently.

Immunohistochemically stained slides were scanned using a NanoZoomer 2.0 HT (Hamamatsu Photonics K.K., Hamamatsu City, Japan) with a 20× objective magnification (0.5 µm resolution). The captured digital images were analyzed using Visiopharm software (version 4.5.1.324; Visiopharm, Hørsholm, Denmark). The intensity of brown staining (negative, intensity < weak; positive, intensity > weak) was obtained using a predefined algorithm and optimized settings. The overall immunohistochemical score was calculated by multiplying the staining intensity by the percentage of positively stained cells.

### 2.3. Public Databases

mRNA levels of Nectin-1, -3, and -4 genes in normal and EC tissues from the cteru2 dataset (Oncomine; Compendia Bioscience, https://www.oncomine.org, accessed on 27 November 2021) were obtained. No further normalization of the derived beta values was performed. Alteration frequency of Nectin-4, MLH1, MSH2, MSH6, and PMS2 genes and the correlations between these genes were determined by analyzing The Cancer Genome Atlas (TCGA) PanCancer Altas studies and gene status, especially whether they were amplified, using the cBioPortal for cancer genomics (http://www.cbioportal.org, accessed on 28 January 2022).

### 2.4. Statistical Analysis

The Mann–Whitney U test was used to compare protein expression levels between groups. For survival analysis, expression values were dichotomized (high vs. low), with cutoff values showing the most discriminative power (3.258 for Nectin-4). Survival distributions were estimated using the Kaplan–Meier method with a log-rank test. A Cox multivariate proportional hazards model was used to identify independent predictors of survival. Statistical analysis was performed using SPSS version 23.0 (SPSS, Chicago, IL, USA) and GraphPad Prism 7 (GraphPad Software, Inc., La Jolla, CA, USA). Results are expressed as the mean ± SD based on the control. Receiver operating characteristic (ROC) curve analysis was performed using MedCalc statistical software version 20.019 (MedCalc Software Ltd., Ostend, Belgium). In all cases, *p*-values < 0.05 were considered statistically significant.

## 3. Results

### 3.1. Nectin-4 Is Overexpressed in EC Tissues Compared with Normal Endometrial and EIN Tissues

**Figure 1:**
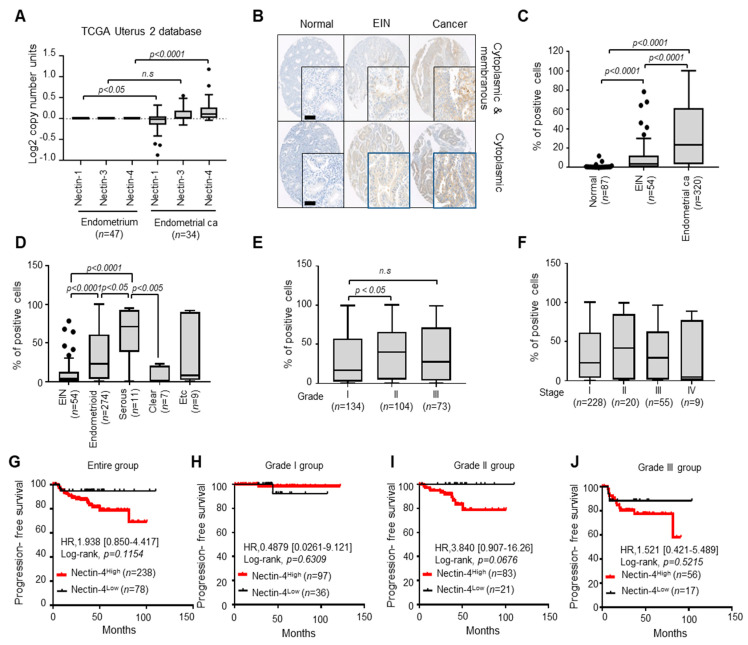
Nectin-4 is overexpressed in endometrial cancer tissues. (**A**) Comparison of the expression of nectin family genes between EC and endometrium tissue. Data on the mRNA expression of Nectin-1, -3, and -4 were obtained from the Oncomine database (http://www.oncomine.com). The Mann–Whitney U test was used to evaluate statistical significance. (**B**,**C**) Representative images of immunohistochemical staining of Nectin-4 expression in tissues from patients with EC and EIN and nonadjacent normal tissue ((**B**); Scale bar: 250 μm). A box plot depicting immunohistochemical staining (**C**). The intensity of brown staining (negative, intensity < weak; positive, intensity > weak) was obtained using a predefined algorithm and optimized settings. The overall immunohistochemical score was calculated by multiplying the staining intensity by the percentage of positively stained cells. The Mann–Whitney U test was used to compare protein expression levels between groups. (**D**) Box and whisker plots showing Nectin-4 expression in tissues from patients with different histological subtypes of EC. A one-way ANOVA test was used to evaluate statistical significance. (**E**,**F**) Box and whisker plots showing Nectin-4 expression according to grade (**E**) and FIGO stage (**F**) in the tissues of patients with ECs. A one-way ANOVA test was used to evaluate statistical significance. (**G**–**I**) Kaplan–Meier curves were used to evaluate the progression-free survival (PFS) in patients with entire (**G**), Grade I (**H**), and Grade II/III (**I**,**J**) ECs. Patients were classified based on high (Nectin-4^High^; >25th percentile) and low (Nectin-4^Low^; <25th percentile) Nectin-4 expression. HR, hazard ratio; 95% CI, 95% confidence interval; *n*, number of patients.

To elucidate the association between the nectin family (Nectin-1,-3, and -4) and EC, we first analyzed the expression of nectin isoforms in EC in a public database (TCGA Uterus2 dataset). Among them, only the copy number of *Nectin-4* was markedly elevated in EC compared with that in the endometrium (Figure 1A). Next, we assessed the clinical relevance of Nectin-4 in archival tumor tissues from patients with EC (*n* = 320) and EIN (*n* = 54) and nonadjacent normal tissue (*n* = 87) using immunohistochemical staining. Detailed clinicopathological characteristics are shown in Table 1. Strong Nectin-4 expression was found in the cytoplasm and membrane of EC tissues compared with that in normal and EIN tissues (Figure 1B). Immunohistochemistry scoring was performed using automated digital image analysis. Box plot analysis revealed that Nectin-4 expression increased with tumor progression from normal epithelium to EC tissues (Figure 1C), with almost no detection in nonadjacent normal tissue (Figure 1B,C). Our histological-type analysis showed strong Nectin-4 expression in serous and endometrioid EC tissues (Figure 1D) compared with that in EIN tissues. No significant expression was observed in clear-cell EC.

We assessed the implication of Nectin-4 expression via clinicopathological parameters in patients with EC. Box plot analysis revealed that Nectin-4 expression levels were associated with patients with Grade II EC compared with those with Grade I EC (Figure 1E; *p* < 0.05). There were no differences in the association between Nectin-4 expression and EC stage (Figure 1F). These results suggest that Nectin-4 is overexpressed in EC and is correlated with high tumor grade.

We examined the relationship between Nectin-4 expression and PFS outcomes. Patients with EC were categorized into high- (Nectin-4^High^) and low-expression (Nectin-4^Low^) groups according to their 25^th^ percentile cutoffs (cutoff value, 3.258%) of the percentage of Nectin-4-positive cells. The Kaplan–Meier plots revealed that the Nectin-4^High^ group showed a shorter PFS than that of the Nectin-4^Low^ group in the entire EC, but there was no statistical significance (Figure 1G; HR = 1.938, *p* = 0.1154). The Nectin-4^High^ group in Grade I EC showed no difference in PFS period compared with the Nectin-4^Low^ group (Figure 1H). In Grade II and III patients with EC, the Nectin-4^High^ group exhibited a shorter PFS than that of the Nectin-4^Low^ group (Figure 1I,J; HR = 3.840, HR = 1.521; *p* = 0.0676, *p* = 0.5215, respectively), but there was no statistical significance. These results suggest that high Nectin-4 expression may be associated with short PFS in Grade II and III EC. Altogether, our results suggest that Nectin-4 is overexpressed in EC, correlates with high-grade tumors, and is associated with the aggressive behavior of EC.

### 3.2. Nectin-4 Is a Powerful Diagnostic Marker of EC

**Figure 2:**
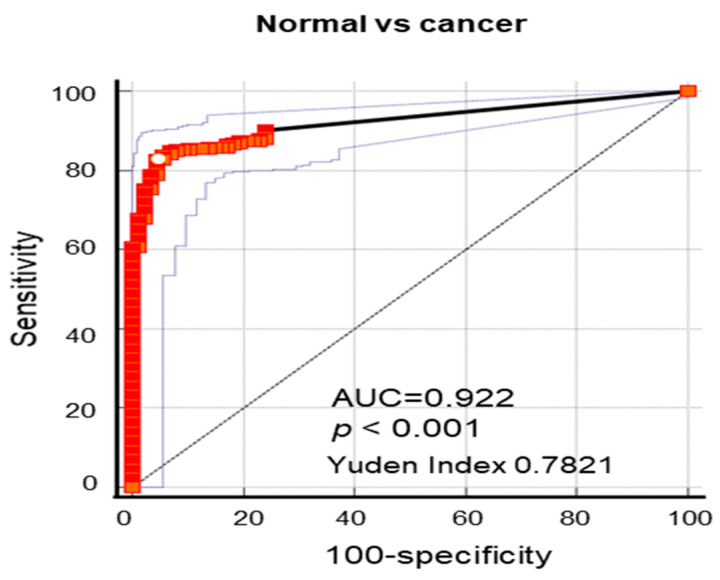
Receiver operating characteristic (ROC) curves of the promising endometrial cancer (EC) diagnostic biomarker. ROC curves were used to confirm the diagnostic value of Nectin-4 expression for identifying EC.

To investigate the diagnostic value of Nectin-4 in EC, its relevance was assessed using ROC curve analysis. The ROC curve showed that patients with EC could be distinguished from those with normal epithelium according to the optimal cutoff value of 0.7822 of Nectin-4-positive cells, with an area under the curve (AUC) value of 0.922 (95% CI, 0.895–0.946) (Figure 2, Table 2). Nectin-4 had higher specificity (95.40%) and sensitivity (82.81%) with high positive predictive value (PPV; 98.5%) and negative predictive value (NPV; 60.1%) (Table 2), indicating that Nectin-4 is a superior diagnostic EC marker. Next, we calculated the odds ratio (OR), which defines the relationship between potential biomarkers and EC diagnosis. The OR was 79.01 (95% CI = 30.60–203.99, *p* < 0.0001) for EC tissues (Table 3). These data demonstrate that Nectin-4 is a potential diagnostic and screening EC marker.

### 3.3. Nectin-4 Is Associated with MMR Deficiency

**Figure 3:**
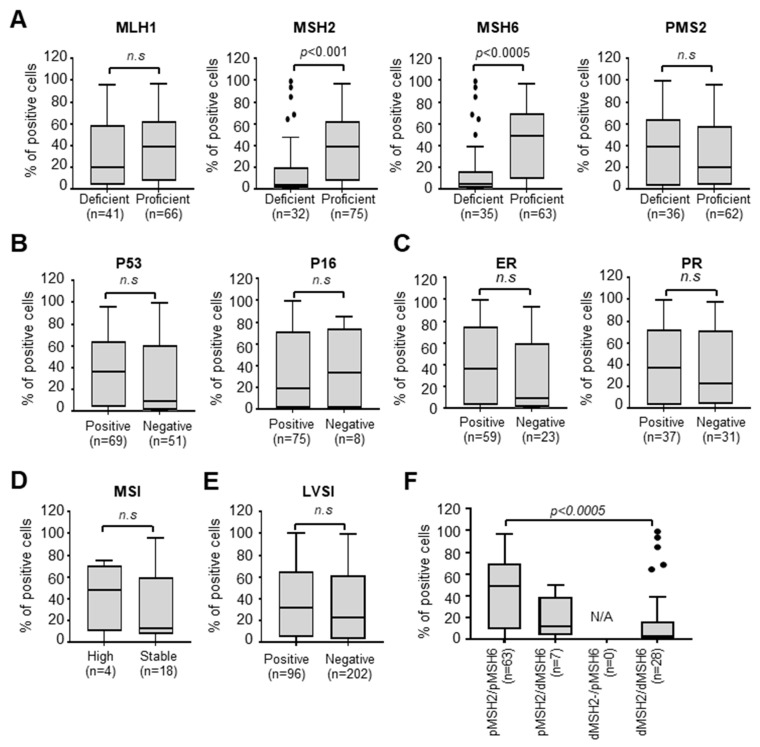
MSH2- and MSH6-deficient EC tissues display impaired Nectin-4 expression. (**A**–**E**). Box plot analysis showing Nectin-4 expression via expression status of MMR protein (**A**), p53 (**B**), hormone receptor (**C**), MSI (**D**), and LVSI (**E**) using immunohistochemistry. The Mann–Whitney U test was used to evaluate statistical significance. (**F**) Box plot analysis showing Nectin-4 expression via the status of MSH2 and/or MSH6 in tissues from patients with EC.

To elucidate the relationship between Nectin-4 and EC risk factors, including genetic factors, we analyzed MMR deficiency-related genes (*MLH1*, *MSH2*, *MSH6*, and *PMS2*), p53, p16, hormone receptor status, and lymphovascular space invasion (LVSI). Box plot analysis revealed that compared with MSH2- or MSH6-proficient patients, MSH2- or MSH6-deficient patients with EC showed impaired Nectin-4 expression, whereas *MLH1* and *PMS2* status did not affect Nectin-4 expression (Figure 3A). There were no differences between Nectin-4 expression and other factors, including p53, p16, ER/PR status, microsatellite instability (MSI), and LSVI (Figure 3B–E). When both MSH2 and MSH6 were deficient, Nectin-4-positive cells were fewer than when both MSH2 and MSH6 were proficient (Figure 3F). These results suggest that Nectin-4 expression is correlated with *MSH2/MSH6* deficiency in EC.

### 3.4. Alteration Frequency of MMR Is Associated with Nectin-4 Expression in EC

**Figure 4:**
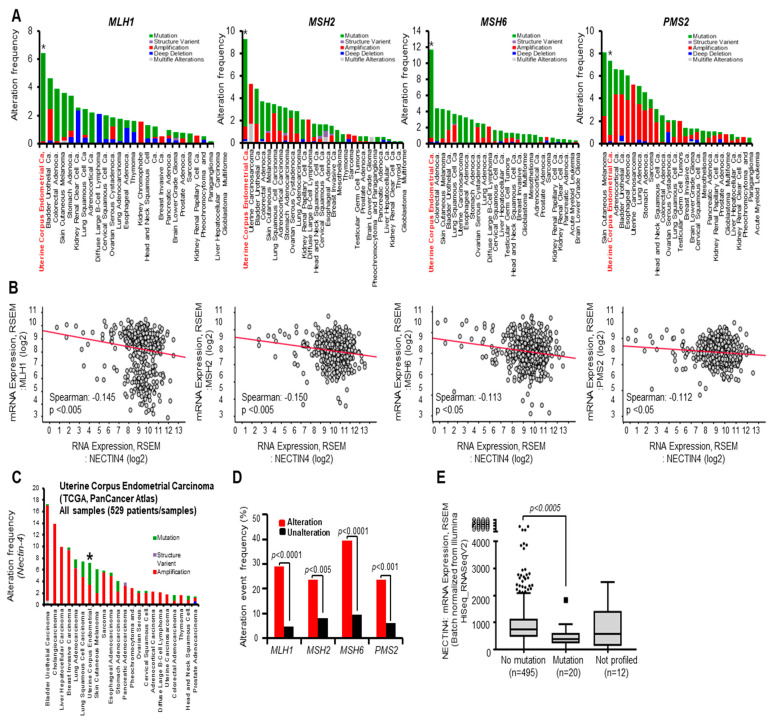
Alteration frequency of MMR is associated with Nectin-4 expression in EC. (**A**) Gene alteration frequency of MMR proteins in TCGA PanCancer Atlas Studies was analyzed using the cBioPortal tool. Uterine corpus endometrial cancers is marked with * (**B**) Correlation between mRNA expression of Nectin-4 and MMR genes in endometrial cancer (EC) tumors from TCGA PanCancer Atlas Studies using the cBioPortal tool. The expression value is presented as the Z-score fold-change in RNA-seq expression (v2 RSEM). The Spearman’s co-relation rank for each comparison is shown, and significance is shown using the *p*-value. (**C**) Gene alteration frequency of Nectin-4 proteins in TCGA PanCancer Atlas Studies was analyzed using the cBioPortal tool. (**D**) Mutation frequency (%) of Nectin-4 in the altered and unaltered groups of MMR in patients with EC. Data were analyzed using the cBioPortal tool. (**E**) Correlation between mRNA expression and mutation of Nectin-4.

To elucidate the alterations in MMR in EC, we assessed the alteration frequency of MMR in patients with EC from TCGA. Our bioinformatics analysis using a public database revealed that alterations in genes, such as *MLH1*, *MSH2*, *MSH6*, and *PMS2*, are the strongest in EC among various cancer types (Figure 4A). We found that Nectin-4 expression negatively correlated with the expression of *MLH1*, *MSH2*, *MSH6*, and *PMS2* in EC (Figure 4B). Notably, the alteration frequency of Nectin-4 was 8% in EC (Figure 4C; mutation, 4%; structure variant, 1%; amplification, 4%). Next, to confirm the relationship between mutant Nectin-4 and the alteration of MMR genes, we analyzed the alteration frequency of the Nectin-4 group via the MMR status in EC (TCGA, PanCancer Atlas). The mutation frequency of Nectin-4 was higher than that of the unaltered MMR gene group (Figure 4D). Furthermore, we observed significantly lower Nectin-4 mRNA expression in the group with Nectin-4 mutation than that in the group without Nectin-4 mutation, which was statistically significant (Figure 4E; *p* < 0.0005).

Finally, EC data from TCGA were analyzed to obtain tumor mutational burden (TMB) and MSI profiles. We assessed the correlation between the TMB and MSI sensor score and the alteration frequency of Nectin-4, MSH2, and MSH6. EC tumors with alterations in MSH2 and MSH6 had higher MSI (median; 2.1 vs. 0.09, 2.6 vs. 0.0, respectively) and TMB (median; 166.9 vs. 1.5, 192.3 vs. 1.5, respectively) rates compared with those of the unaltered group. The Nectin-4 alteration groups showed higher MSI (median; 0.97 vs. 0.09) and TMB (median; 15.4 vs. 1.5) proportions than those of the unaltered groups (Table 4). Thus, these data suggest that Nectin-4 alteration is associated with TMB and MSI scores.

### 3.5. High Expression of Nectin-4 in MSH2 Deficiency Caused a Short PFS in EC

**Figure 5:**
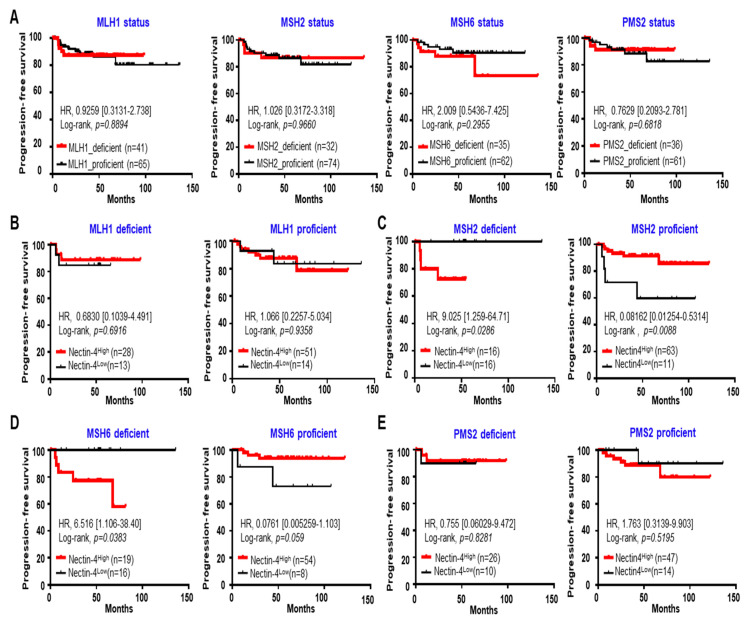
High expression of Nectin-4 in MSH2-deficient patients with EC is correlated with a short progression-free survival (PFS). (**A**) Kaplan-Meier curves were used to evaluate PFS in patients with EC, and the MMR status was determined using immunohistochemistry. (**B**–**E**) Kaplan–Meier curves were used to determine the effects of PFS on the MLH1 (**B**), MSH2 (**C**), MSH6 (**D**), and PMS2 (**E**) statuses in patients. Patients were classified into high (>25^th^ percentile) and low (<25^th^ percentile) Nectin-4 expression groups according to their 25^th^ percentile of Nectin-4 expression. HR, hazard ratio; 95% CI, 95% confidence interval; *n*, number of patients.

We examined the relationship between Nectin-4 expression levels and PFS. First, we monitored the effect of PFS via the statuses of MSH1, MSH2, MSH6, and PMS2. MMR status did not affect the PFS period in patients with EC (Figure 5A). The patients were categorized into high- (Nectin-4^High^) and low-expression (Nectin-4^Low^) groups according to their 25^th^ percentile cutoffs (3.58%). Kaplan–Meier plots revealed that patients with MSH2 deficiency in the Nectin-4^High^ groups exhibited a shorter PFS period (HR = 9.025, *p* = 0.0286) than those in the Nectin-4^Low^ group. By contrast, patients with MSH2 proficiency in the Nectin-4^High^ group exhibited a longer PFS period (HR = 0.0816, *p* = 0.0088) than those in the Nectin-4^Low^ group (Figure 5B–E). Similar results were obtained for MSH6 (Figure 5B–E). However, Nectin-4 expression did not affect PFS in patients with MLH1 and PMS2 deficiency or proficiency (Figure 5B–E). Detailed clinicopathological characteristics are shown in Table 5. These results suggest that high expression of Nectin-4 is a predictive marker for poor prognosis in MSH2/6-deficient patients with EC.

### 3.6. Increase in LVSI-Positive Patients in the MSH2-Deficient/Nectin-4^High^ Group

**Figure 6:**
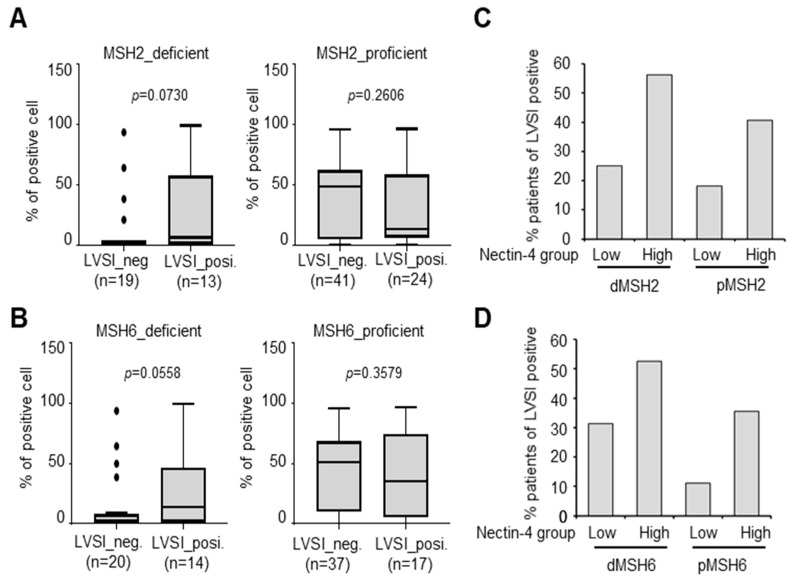
LVSI-positive patients increase in MSH2-deficient/Nectin-4^High^ groups. (**A**,**B**) Percentage of patients with LVSI-positive MSH2- (**A**) and MSH6-deficient (**B**) EC. (**C**,**D**) Percentage of LVSI-positive patients in MSH2- (**C**) and MSH6-deficient (**D**) EC. The Mann–Whitney U test was used to evaluate statistical significance (one-tailed test).

To elucidate the correlation between LVSI and short PFS in MSH2- and MSH6-deficient/Nectin4^High^ groups, we evaluated LVSI-positive cells to determine the effect of high Nectin-4 expression on MSH2- and MSH6-deficient patients with EC. MSH2 or MSH6 deficiency did not affect the proportion of LVSI-positive cells (Figure 6A,B). Patients in the Nectin-4^High^ group were more likely to have LVSI than those with MSH2-proficient tumors (65.4% vs. 53.4%) (Figure 6C, Table 6). In these groups, the advanced-stage population is higher than the MSH2-proficient/Nectin4^High^ group (Table 7). Similar results were obtained for MSH6 (Figure 6D). These results suggest that the number of LVSI-positive patients increased in the Nectin-4^High^ group, indicating that Nectin-4 overexpression is a poor prognostic marker in MSH2- and MSH6-deficient patients with EC.

### 3.7. Decrease in CD8-Positive Patients in the MSH2-Deficient/Nectin-4^High^ Group

Because MSI/MMR status is the common denominator for immunotherapy treatment of patients with several solid tumors, we analyzed the ratio of CD8-positive patients with MSH-deficient EC in Nectin-4^High^ and Nectin-4^Low^ groups (Table S2). The population of MSH2-deficient patients with CD8-positive cells was 13.6% compared with 86.4% of their MSH2-proficient counterparts. Similar results were observed in MSH6-deficient and MSH6-proficient patients with EC. The percentage of CD8-positive patients in the MSH2-deficient/Nectin-4^High^ group was lower than that in the MSH2-deficient/Nectin-4^Low^ group (16.7% vs. 83.3%, respectively) and that in the MSH2-proficient/Nectin-4^High^ group was higher than that in the MSH2-proficient/Nectin-4^Low^ group (78.9% vs. 21.2%, respectively). Similar results were obtained in the MSH6 group. These results suggest the possibility that MSH-dependent Nectin-4 expression is associated with different levels of infiltrating CD8-positive cell migration in ECs.

## 4. Discussion

DNA MMR impairment occurs in EC; however, its therapeutic implications remain uncertain. In this study, we demonstrate that Nectin-4 is a good diagnostic and prognostic marker in EC. Furthermore, we highlighted that high expression of Nectin-4 in MSH2- or MSH6-deficient patients with EC indicated short PFS as a poor predictive marker in EC. Compelling evidence has emerged in recent years supporting a new tumor biomarker, Nectin-4, in several different carcinomas [21,22,23,24,26,31,32,33]. The expression level of Nectin-4 is significantly associated with cancer cell differentiation, lymph node metastasis, advanced TNM stage, and poor patient prognosis [34]. Abnormal expression of both membranous and soluble forms of Nectin-4 has been found in human breast cancer tissues and sera, and the levels of both forms of Nectin-4 can be used as important biomarkers and prognostic predictors in patients with breast cancer [29,34,35]. However, few studies have shown that Nectin-4 expression is a good prognostic biomarker [21,30,36], while accumulating studies have shown that Nectin-4 plays a critical role in tumorigenesis [37,38,39] and lymphangiogenesis [40]. Furthermore, Nectin-4 as a stem cell marker induces WNT/beta-catenin signaling via the PI3K/Akt axis [27]. Therefore, Nectin-4-targeted therapy is a potent strategy for treating cancers with high Nectin-4 expression. Recently, enfortumab vedotin, an ADC targeting Nectin-4, has been approved for treating patients with bladder cancer with Nectin-4 overexpression [31]. However, despite the recent evidence of Nectin-4 overexpression in cancer and its therapeutic efficacy, the biological significance of Nectin-4 in EC and its clinical potential value as a therapeutic target remain unknown. Here, we found a high expression of Nectin-4 in EC, whereas it was not expressed in the adjacent normal endometrium. ROC analysis showed that a cutoff value of 0.782 for Nectin-4-positive cells displayed a high AUC (>0.922) with high specificity and sensitivity, suggesting that high Nectin-4 expression can help distinguish EC tissues and nonadjacent normal tissue. Thus, Nectin-4 is a novel diagnostic tool and screening marker for assessing EC and can enhance the accuracy of the methods used to predict high-risk EC.

EC is currently being studied using a new molecular biological approach in addition to the traditional cytopathological classification [41,42]. TCGA (2013) proposed the EC subtypes as ProMisE (Proactive Molecular Risk Classifier for Endometrial Cancer), which includes POLE, MMRd, p53 wild-type/copy-number-low, and p53-mutated/copy-number-high [43]. This molecular categorization leads to better prediction of clinical outcomes than morphology and traditional risk parameters, such as stage and the presence of LVSI [15]. However, there are several conflicting reports on the association between MMR status and clinical outcomes [15,44]. In the present study, although we observed the highest frequency of alteration in MMR genes (*MLH1*, *MSH2*, and *MSH6*), we failed to observe different PFS periods according to MMR status. Therefore, the clinical value of MMR status in predicting poor prognosis in patients with EC is urgently required. In the current study, patients with Nectin-4 overexpression in ECs and MSH2/6 deficiency showed short PFS, whereas patients with Nectin-4 overexpression in MSH2/6 proficient EC showed long PFS; these results suggest the differential prognostic diagnostic value for patients with Nectin-4 overexpression according to MSH2/6 status. However, in the case of other MMR proteins, such as MLH1 and PMS2, overexpression of Nectin-4 did not affect PFS. Therefore, our results suggest that high expression of Nectin-4 in patients with EC and MSH2 and MSH6 deficiency may help improve the accuracy of EC diagnosis and prognosis.

MSI, a mutation induced by errors in the DNA replication process, may occur due to the loss of function of the MMR gene. MSI and MMR have important diagnostic and prognostic implications in colon and gastric cancers and ECs [45]. Approximately 30% of primary ECs are MSI-high/hypermutated (MSI-H), and 13–30% of recurrent ECs are MSI-H or MMR protein-deficient (dMMR) [46]. Consistent with these reports, our TMB and MSI profiles showed a high MSI sensor score and TMB in the alteration frequency of MSH2 and MSH6. Importantly, Nectin-4 gene alteration groups displayed a high MSI sensor score and TMB. Thus, we speculated that there is a correlation between MSI, MMR, and Nectin-4 expression. Recently, MSI-H/dMMR solid tumors have been considered immunotherapy biomarkers [47]. CD8+, Th1, Th2, follicular helper T cells, and T cell markers are significantly higher in patients with MSI-H/dMMR colorectal cancer than in microsatellite stable (MSS)/MMR protein-proficient (pMMR) colorectal cancer [48]. Notably, we found that the number of patients with CD8-positive tumor-infiltrating lymphocytes in Nectin-4^High^/MSH-deficient groups is lesser than that in Nectin-4^Low^/MSH-deficient groups. These results suggest that Nectin-4 expression in MSH2/6-deficient EC is associated with different infiltrating CD8+ lymphocytes in ECs. Thus, we speculate that the short PFS observed in the Nectin-4^High^/MSH2/6-deficient groups may correlate with CD8+ lymphocyte recruitment in EC.

Our study has several limitations regarding the mechanism underlying the aggressive role of Nectin-4 in MSH2- and MSH6-deficient EC, despite observing a correlation between short PFS and the MSH2/6-deficient/Nectin4^High^ groups. Although several studies suggest that the regulation of Nectin-4 expression may be mediated by various transcription factors, such as ERRα [49] and SP1 [50], the factors modulating Nectin-4 expression in MSH2/6-deficient ECs remain unclear. Otherwise, a possible mechanism could be crosstalk between Nectin-4 and the DNA repair system. Nectin-4 cis interacts with many cell surface membrane receptors, including growth factor receptors [51,52], hormone receptors [53], and integrins [54]. Nectin-4 activates the PI3K-AKT signaling pathway for DNA synthesis after cis-interaction with ErbB2, enhancing its homodimerization and activation [55]. Our public data analysis revealed that Nectin-4 expression positively correlated with ERBB2 and ERBB3 expression in patients with EC (Figure S1; Spearman’s correlation = 0.203, 0.381; *p* < 0.0001), suggesting that Nectin-4 plays an important role in DNA repair and synthesis in MSH2- and MSH6-deficient ECs against aggressive behavior. However, further studies are required to determine whether this mechanism is responsible for the aggressive behavior of MSH-deficient ECs.

## 5. Conclusions

We demonstrated Nectin-4 as an exceptional diagnostic marker for EC. In addition, we confirmed that in patients with EC and MSH2 or MSH6 deficiency, high levels of Nectin-4 expression predict poor prognosis. Furthermore, the decreased amount of CD8+ tumor-infiltrating lymphocytes in patients with EC and MSH deficiency or Nectin-4 overexpression is thought to provide important clues for screening and monitoring patients responding to cancer immunotherapy. However, further studies are required to fully understand the role and clinical significance of Nectin-4 in EC and its potential as a biomarker for diagnosis and treatment.

## Figures and Tables

**Table 1:** Correlation between Nectin-4 expression and clinicopathological characteristics of patients with endometrial cancer.

Characteristic	Nectin-4 Expression		
	Nectin-4^High^	Nectin-4^Low^	*p*-Value
	*n* (%)	*n* (%)	
Diagnosis			0.000 *
Nonadjacent normal tissue	2 (2.3%)	85 (97.7%)	
EIN	28 (51.9%)	26 (48.1%)	
Cancer	240 (75.0%)	80 (25.0%)	
Age			0.098
≥50	179 (74.6%)	52 (65.0%)	
50<	61 (25.4%)	28 (35.0%)	
FIGO stage			0.900
I	173 (72.1%)	55 (68.8%)	
II	14 (5.8%)	6 (7.5%)	
III	40 (16.7%)	15 (18.8%)	
IV	6 (2.5%)	3 (3.8%)	
Recurrence	6 (2.5%)	1 (1.3%)	
Unclassified	1 (0.4%)	0 (0%)	
Differentiation			0.059
Grade I	97 (40.4%)	37 (46.3%)	
Grade II	83 (34.6%)	21 (26.3%)	
Grade III	56 (23.3%)	17 (21.3%)	
Mix	0 (0%)	2 (2.5%)	
Unclassified	4 (1.7%)	3 (3.8%)	
Histology			0.076
Endometrioid	206 (85.8%)	68 (85.0%)	
Serous	10 (4.2%)	1 (1.3%)	
Clear	2 (0.8%)	5 (6.3%)	
Mix	14 (5.8%)	4 (5.0%)	
ETS	7 (2.9%)	2 (2.5%)	
Unclassified	1 (0.4%)	0 (0.0%)	
CA125 (U/mL)			0.559
≥35	167 (69.6%)	58 (72.5%)	
35<	60 (25.0%)	20 (25.0%)	
Unclassified	13 (5.4%)	2 (2.5%)	
MSH2 status			0.001 *
Deficient	16 (6.7%)	16 (20.0%)	
Proficient	63 (26.3%)	12 (15.0%)	
Unclassified	161 (67.1%)	52 (65.0%)	
MSH6 status			0.003 *
Deficient	19 (7.90%)	16 (20.0%)	
Proficient	54 (22.5%)	9 (11.3%)	
Unclassified	167 (69.6%)	55 (68.8%)	

*n*, number of patients; N/A, not applicable; FIGO, Federation of Gynecology and Obstetrics. * *p* < 0.05.

**Table 2:** ROC curve analysis for Nectin-4 in patients with endometrial cancer.

Group	Youden Index J	ROC–AUC (95%CI)	Sensitivity (95%CI)	Specificity (95%CI)	PPV (95%CI)	NPV (95%CI)
Nectin-4	0.7821	0.922 (0.892–0.946)	82.81 (78.2–86.8)	95.40 (88.6–98.7)	98.5 (96.2–99.4)	60.1 (54.2–65.8)

ROC–AUC, receiver operating characteristics–area under the curve; PPV, positive predictive value; NPV, negative predictive value.

**Table 3:** Odds ratios for endometrial cancer risk of Nectin-4.

Group	Odds Ratio	(95% CI)	*p*-Value
Nectin-4 > 0.7821%	79.01	(30.60–203.99)	<0.0001

**Table 4:** Correlation between tumor mutational burden and MSI sensor score via the alteration frequency of Nectin-4, MSH2, and MSH6.

Gene		Altered Group					Unaltered Group					
		Max.	75%	Median	25%	Min.	Max.	75%	Median	25%	Min.	*p*-Value
Nectin-4	Tumor mutation burden (nonsynonymous)	748.4	300.6	15.4	2.1	0.9	38.7	16.4	2.4	1.5	0	<0.0001
	MSIsensor Score	23.17	9.49	0.97	0.37	0	26.4	10.65	0.41	0.09	0	0.0431
MSH2	Tumor mutation burden (nonsynonymous)	758.8	325.1	166.9	36.1	1.1	33.0	14.1	2.3	1.5	0	<0.0001
	MSIsensor Score	35.7	16.0	2.1	0.35	0.0	24.4	9.83	0.4	0.09	0	<0.0001
MSH6	Tumor mutation burden (nonsynonymous)	763.9	325.1	192.3	32.6	1.1	27.1	11.7	2.2	1.5	0	<0.0001
	MSIsensor Score	34.8	14.3	2.6	0.7	0	22.8	9.1	0.3	0.0	0	<0.0001

**Table 5:** Expression of Nectin-4 in relation to the clinicopathological characteristics via MSH2/6 status of endometrial cancer patients.

Clinicopathologic Variables	MSH2 Status			MSH6 Status		
	Deficiency	Proficiency	*p*-Value	Deficiency	Proficiency	*p*-Value
	*n* = 32	*n* = 75		*n* = 35	*n* = 63	
Age			0.091			0.604
≥50	20 (62.5%)	58 (77.3%)		25 (71.4%)	48 (76.2%)	
<50	12 (37.5%)	17 (22.7%)		10 (28.6%)	15 (23.8%)	
FIGO stage			0.077			0.317
I/II	23 (71.9%)	54 (72.0%)		23 (65.7%)	50 (79.4%)	
III/IV	9 (28.1%)	14 (18.7%)		10 (28.6%)	9 (14.3%)	
Recurrence	0 (0%)	6 (8.0%)		2 (5.7%)	3 (4.8 %)	
Unclassified	0 (0%)	1 (1.3 %)		0 (0 %)	1 (1.6%)	
Differentiation			0.580			0.335
Grade I	10 (31.3%)	28 (37.3%)		11(31.4%)	27 (42.9%)	
Grade II/III	21 (65.6%)	42 (56.0%)		21 (60.0 %)	34 (54.0%)	
Unclassified	1 (3.1 %)	5 (6.7%)		3 (8.6 %)	2 (3.2%)	
CA125 (U/mL)			0.493			0.739
≥35	11 (34.4%)	27 (36.0%)		13 (37.1 %)	20 (31.7%)	
<35	20 (62.5%)	41 (54.7%)		20 (57.1%)	37 (58.7%)	
Unclassified	1 (3.1%)	7 (9.3%)		2 (5.7%)	6 (9.5%)	
Lymphovascular invasion			0.089			0.129
Absent	19 (59.4%)	41 (54.7%)		20 (57.1%)	37 (58.7%)	
Present	13 (40.6 %)	24 (32.0 %)		14 (40.0%)	17 (27.0%)	
Unclassified	0 (0%)	10 (13.3%)		1 (2.9%)	9 (14.3%)	
Nectin-4 expression			0.000 *			0.001 *
High	16 (50%)	63 (84.0%)		19 (54.3%)	54 (85.7%)	
Low	16 (50%)	12 (16.0%)		16 (45.7%)	9 (14.3%)	

Chi-square test was used to test the association between clinicopathological parameters and the status of MSH2 and MSH6. * *p* < 0.05.

**Table 6:** Percentage of LVSI-positive patients along Nectin-4 expression pattern in MSH2/6-deficient EC.

Groups		LVSI-Positive %	LVSI-Negative %	Total	*p*-Value
MSH2_deficient	Nectin-4^High^	56.3% (9/16)	43.8% (7/16)	16	0.072
	Nectin-4^Low^	25% (4/16)	75% (12/16)	16	
MSH2_proficient	Nectin-4^High^	40.7% (22/54)	59.3% (32/54)	54	0.158
	Nectin-4^Low^	18.2% (2/11)	81.8% (9/11)	11	
MSH6_deficient	Nectin-4^High^	52.6% (10/19)	47.4% (9/19)	19	0.203
	Nectin-4^Low^	31.3% (5/16)	68.8% (11/16)	16	
MSH6_proficient	Nectin-4^High^	35.6% (16/45)	64.4% (29/45)	45	0.149
	Nectin-4^Low^	11.1% (1/9)	88.9% (8/9)	9	

**Table 7:** Correlation between advanced stage and Nectin-4 expression in MSH2-deficient EC patients.

Clinicopathologic Variables	MSH2 Deficient			MSH2 Proficient		
	Nectin-4^High^	Nectin-4^Low^	*p*-Value	Nectin-4^High^	Nectin-4^Low^	*p*-Value
	*n* = 16	*n* = 16		*n* = 57	*n* = 11	
FIGO stage			0.694			0.158
I/II	11 (68.8%)	12 (75.0%)		47 (82.5%)	7 (63.6%)	
III/IV	5 (31.3%)	4 (25.0%)		10 (17.5%)	4 (36.4%)	

Chi-square test was used to test the association between advanced stage and Nectin-4 expression in MSH2-deficient EC patients.

## Data Availability

All data generated or analyzed during this study are included in the manuscript. Further inquiries should be directed to the corresponding author.

## Supplementary Materials

The following supporting information can be downloaded at: https://www.mdpi.com/article/10.3390/cancers15102865/s1, Figure S1: Alteration frequency of ERBB2 and ERBB3 is associated with Nectin-4 expression in EC; Table S1: Inclusion and exclusion criteria for retrospective study; Table S2: Ratio of groups of Nectin-4^High^ and Nectin-4^Low^ in CD8-positive patients with MSH-deficient EC.

## References

[1] Parkin D.M., Pisani P., Ferlay J. (1999). Estimates of the worldwide incidence of 25 major cancers in 1990. Int. J. Cancer.

[2] Bray F., Ferlay J., Soerjomataram I., Siegel R.L., Torre L.A., Jemal A. (2018). Global cancer statistics 2018: GLOBOCAN estimates of incidence and mortality worldwide for 36 cancers in 185 countries. CA Cancer J. Clin..

[3] Masuda K., Banno K., Yanokura M., Kobayashi Y., Kisu I., Ueki A., Ono A., Asahara N., Nomura H., Hirasawa A. (2011). Relationship between DNA Mismatch Repair Deficiency and Endometrial Cancer. Mol. Biol. Int..

[4] Aarnio M., Sankila R., Pukkala E., Salovaara R., Aaltonen L.A., de la Chapelle A., Peltomäki P., Mecklin J.P., Järvinen H.J. (1999). Cancer risk in mutation carriers of DNA-mismatch-repair genes. Int. J. Cancer.

[5] MacDonald N.D., Salvesen H.B., Ryan A., Iversen O.E., Akslen L.A., Jacobs I.J. (2000). Frequency and prognostic impact of microsatellite instability in a large population-based study of endometrial carcinomas. Cancer Res..

[6] Bell D.W., Ellenson L.H. (2019). Molecular genetics of endometrial carcinoma. Annu. Rev. Pathol..

[7] Reitsam N.G., Märkl B., Dintner S., Waidhauser J., Vlasenko D., Grosser B. (2022). Concurrent loss of MLH1, PMS2 and MSH6 immunoexpression in digestive system cancers indicating a widespread dysregulation in DNA repair processes. Front. Oncol..

[8] McCoy P., Mangiola S., Macintyre G., Hutchinson R., Tran B., Pope B., Georgeson P., Hong M.K.H., Kurganovs N., Lunke S. (2021). MSH2-deficient prostate tumours have a distinct immune response and clinical outcome compared to MSH2-deficient colorectal or endometrial cancer. Prostate Cancer Prostatic. Dis..

[9] McMeekin D.S., Tritchler D.L., Cohn D.E., Mutch D.G., Lankes H.A., Geller M.A., Powell M.A., Backes F.J., Landrum L.M., Zaino R. (2016). Clinicopathologic Significance of Mismatch Repair Defects in Endometrial Cancer: An NRG Oncology/Gynecologic Oncology Group Study. J. Clin. Oncol..

[10] Shikama A., Minaguchi T., Matsumoto K., Akiyama-Abe A., Nakamura Y., Michikami H., Nakao S., Sakurai M., Ochi H., Onuki M. (2016). Clinicopathologic implications of DNA mismatch repair status in endometrial carcinomas. Gynecol. Oncol..

[11] Cosgrove C.M., Cohn D.E., Hampel H., Frankel W.L., Jones D., McElroy J.P., Suarez A.A., Zhao W., Chen W., Salani R. (2017). Epigenetic silencing of MLH1 in endometrial cancers is associated with larger tumor volume, increased rate of lymph node positivity and reduced recurrence-free survival. Gynecol. Oncol..

[12] Carr C., Son J., Yao M., Priyadarshini A., Marquard J., Vargas R., Michener C., AlHilli M.M. (2020). Clinicopathologic characteristics and outcomes of endometrial Cancer patients with mismatch repair deficiency in the era of universal Lynch syndrome screening. Gynecol. Oncol..

[13] Pasanen A., Loukovaara M., Bützow R. (2020). Clinicopathological significance of deficient DNA mismatch repair and MLH1 promoter methylation in endometrioid endometrial carcinoma. Mod. Pathol..

[14] Kim S.R., Tone A., Kim R.H., Cesari M., Clarke B.A., Eiriksson L., Hart T., Aronson M., Holter S., Lytwyn A. (2021). Understanding the clinical implication of mismatch repair deficiency in endometrioid endometrial cancer through a prospective study. Gynecol. Oncol..

[15] Diaz-Padilla I., Romero N., Amir E., Matias-Guiu X., Vilar E., Muggia F., Garcia-Donas J. (2013). Mismatch repair status and clinical outcome in endometrial cancer: A systematic review and meta-analysis. Crit. Rev. Oncol. Hematol..

[16] Nagle C.M., O’Mara T.A., Tan Y., Buchanan D.D., Obermair A., Blomfield P., Quinn M.A., Webb P.M., Spurdle A.B. (2018). Australian Endometrial Cancer Study Group. Endometrial cancer risk and survival by tumor MMR status. J. Gynecol. Oncol..

[17] Noyce R.S., Richardson C.D. (2012). Nectin 4 is the epithelial cell receptor for measles virus. Trends Microbiol..

[18] Mühlebach M.D., Mateo M., Sinn P.L., Prüfer S., Uhlig K.M., Leonard V.H., Navaratnarajah C.K., Frenzke M., Wong X.X., Sawatsky B. (2011). Adherens junction protein nectin-4 is the epithelial receptor for measles virus. Nature.

[19] Bouleftour W., Guillot A., Magne N. (2022). The Anti-Nectin 4: A Promising Tumor Cells Target. A Systematic Review. Mol. Cancer Ther..

[20] Tomiyama E., Fujita K., Rodriguez Pena M.D.C., Taheri D., Banno E., Kato T., Hatano K., Kawashima A., Ujike T., Uemura M. (2020). Expression of Nectin-4 and PD-L1 in Upper Tract Urothelial Carcinoma. Int. J. Mol. Sci..

[21] Takano A., Ishikawa N., Nishino R., Masuda K., Yasui W., Inai K., Nishimura H., Ito H., Nakayama H., Miyagi Y. (2009). Identification of nectin-4 oncoprotein as a diagnostic and therapeutic target for lung cancer. Cancer Res..

[22] Derycke M.S., Pambuccian S.E., Gilks C.B., Kalloger S.E., Ghidouche A., Lopez M., Bliss R.L., Geller M.A., Argenta P.A., Harrington K.M. (2010). Nectin 4 overexpression in ovarian cancer tissues and serum: Potential role as a serum biomarker. Am. J. Clin. Pathol..

[23] Nishiwada S., Sho M., Yasuda S., Shimada K., Yamato I., Akahori T., Kinoshita S., Nagai M., Konishi N., Nakajima Y. (2015). Nectin-4 expression contributes to tumor proliferation, angiogenesis and patient prognosis in human pancreatic cancer. J. Exp. Clin. Cancer Res..

[24] Zhang Y., Zhang J., Shen Q., Yin W., Huang H., Liu Y., Ni Q. (2018). High expression of Nectin-4 is associated with unfavorable prognosis in gastric cancer. Oncol. Lett..

[25] Zhang Y., Liu S., Wang L., Wu Y., Hao J., Wang Z., Lu W., Wang X.A., Zhang F., Cao Y. (2016). A novel PI3K/AKT signaling axis mediates Nectin-4-induced gallbladder cancer cell proliferation, metastasis and tumor growth. Cancer Lett..

[26] Pavlova N.N., Pallasch C., Elia A.E., Braun C.J., Westbrook T.F., Hemann M., Elledge S.J. (2013). A role for PVRL4-driven cell-cell interactions in tumorigenesis. eLife.

[27] Siddharth S., Goutam K., Das S., Nayak A., Nayak D., Sethy C., Wyatt M.D., Kundu C.N. (2017). Nectin-4 is a breast cancer stem cell marker that induces WNT/β-catenin signaling via Pi3k/Akt axis. Int. J. Biochem. Cell Biol..

[28] Zhang Y., Chen P., Yin W., Ji Y., Shen Q., Ni Q. (2018). Nectin-4 promotes gastric cancer progression via the PI3K/AKT signaling pathway. Hum. Pathol..

[29] Heath E.I., Rosenberg J.E. (2021). The biology and rationale of targeting nectin-4 in urothelial carcinoma. Nat. Rev. Urol..

[30] Fabre-Lafay S., Monville F., Garrido-Urbani S., Berruyer-Pouyet C., Ginestier C., Reymond N., Finetti P., Sauvan R., Adélaïde J., Geneix J. (2007). Nectin-4 is a new histological and serological tumor associated marker for breast cancer. BMC Cancer.

[31] Challita-Eid P.M., Satpayev D., Yang P., An Z., Morrison K., Shostak Y., Raitano A., Nadell R., Liu W., Lortie D.R. (2016). Enfortumab Vedotin Antibody-Drug Conjugate Targeting Nectin-4 Is a Highly Potent Therapeutic Agent in Multiple Preclinical Cancer Models. Cancer Res..

[32] Siegel R., Ma J., Zou Z., Jemal A. (2014). Cancer statistics, 2014. CA Cancer J. Clin..

[33] Moore M.J., Goldstein D., Hamm J., Figer A., Hecht J.R., Gallinger S., Au H.J., Murawa P., Walde D., Wolff R.A. (2007). Erlotinib plus gemcitabine compared with gemcitabine alone in patients with advanced pancreatic cancer: A phase III trial of the National Cancer Institute of Canada Clinical Trials Group. J. Clin. Oncol..

[34] Rajc J., Gugić D., Fröhlich I., Marjanović K., Dumenčić B. (2017). Prognostic role of Nectin-4 expression in luminal B (HER2 negative) breast cancer. Pathol. Res. Pract..

[35] Siddharth S., Nayak A., Das S., Nayak D., Panda J., Wyatt M.D., Kundu C.N. (2018). The soluble nectin-4 ecto-domain promotes breast cancer induced angiogenesis via endothelial Integrin-β4. Int. J. Biochem. Cell Biol..

[36] Zeindler J., Soysal S.D., Piscuoglio S., Ng C.K.Y., Mechera R., Isaak A., Weber W.P., Muenst S., Kurzeder C. (2019). Nectin-4 Expression Is an Independent Prognostic Biomarker and Associated with Better Survival in Triple-Negative Breast Cancer. Front. Med..

[37] Liu Y., Li G., Zhang Y., Li L., Zhang Y., Huang X., Wei X., Zhou P., Liu M., Zhao G. (2022). Nectin-4 promotes osteosarcoma progression and metastasis through activating PI3K/AKT/NF-κB signaling by down-regulation of miR-520c-3p. Cancer Cell Int..

[38] Hashimoto H., Tanaka Y., Murata M., Ito T. (2022). Nectin-4: A Novel Therapeutic Target for Skin Cancers. Curr. Treat Options Oncol..

[39] Kedashiro S., Kameyama T., Mizutani K., Takai Y. (2021). Nectin-4 and p95-ErbB2 cooperatively regulate Hippo signaling-dependent SOX2 gene expression, enhancing anchorage-independent T47D cell proliferation. Sci. Rep..

[40] Sethy C., Goutam K., Das B., Dash S.R., Kundu C.N. (2021). Nectin-4 promotes lymphangiogenesis and lymphatic metastasis in breast cancer by regulating CXCR4-LYVE-1 axis. Vascul. Pharmacol..

[41] Murali R., Soslow R.A., Weigelt B. (2014). Classification of endometrial carcinoma: More than two types. Lancet Oncol..

[42] Alexa M., Hasenburg A., Battista M.J. (2021). The TCGA Molecular Classification of Endometrial Cancer and Its Possible Impact on Adjuvant Treatment Decisions. Cancers.

[43] Talhouk A., McConechy M.K., Leung S., Yang W., Lum A., Senz J., Boyd N., Pike J., Anglesio M., Kwon J.S. (2017). Confirmation of ProMisE: A simple, genomics-based clinical classifier for endometrial cancer. Cancer.

[44] Li K., Luo H., Huang L., Luo H., Zhu X. (2020). Microsatellite instability: A review of what the oncologist should know. Cancer Cell Int..

[45] Kurnit K.C., Westin S.N., Coleman R.L. (2019). Microsatellite instability in endometrial cancer: New purpose for an old test. Cancer.

[46] Zhang X., Wu T., Cai X., Dong J., Xia C., Zhou Y., Ding R., Yang R., Tan J., Zhang L. (2022). Neoadjuvant Immunotherapy for MSI-H/dMMR Locally Advanced Colorectal Cancer: New Strategies and Unveiled Opportunities. Front. Immunol..

[47] Di Dio C., Bogani G., Di Donato V., Cuccu I., Muzii L., Musacchio L., Scambia G., Lorusso D. (2023). The role of immunotherapy in advanced and recurrent MMR deficient and proficient endometrial carcinoma. Gynecol. Oncol..

[48] Gelsomino F., Barbolini M., Spallanzani A., Pugliese G., Cascinu S. (2016). The evolving role of microsatellite instability in colorectal cancer: A review. Cancer Treat Rev..

[49] Wang L., Yang M., Guo X., Yang Z., Liu S., Ji Y., Jin H. (2020). Estrogen-related receptor-α promotes gallbladder cancer development by enhancing the transcription of Nectin-4. Cancer Sci..

[50] Chen J., Bhandari A., Hirachan S., Lv S., Mainali S., Zheng C., Hao R. (2023). A Specificity Protein 1 assists the progression of the papillary thyroid cell line by initiating NECTIN4. Endocr. Metab. Immune Disord. Drug Targets.

[51] Mandai K., Rikitake Y., Mori M., Takai Y. (2015). Nectins and nectin-like molecules in development and disease. Curr. Top. Dev. Biol..

[52] Mizutani K., Kedashiro S., Maruoka M., Ueda Y., Takai Y. (2017). Nectin-like molecule-4/cell adhesion molecule 4 inhibits the ligand-induced dimerization of ErbB3 with ErbB2. Sci. Rep..

[53] Maruoka M., Kedashiro S., Ueda Y., Mizutani K., Takai Y. (2017). Nectin-4 co-stimulates the prolactin receptor by interacting with SOCS1 and inhibiting its activity on the JAK2-STAT5a signaling pathway. J. Biol. Chem..

[54] Sakamoto Y., Ogita H., Hirota T., Kawakatsu T., Fukuyama T., Yasumi M., Kanzaki N., Ozaki M., Takai Y. (2006). Interaction of integrin alpha(v)beta3 with nectin. Implication in cross-talk between cell-matrix and cell-cell junctions. J. Biol. Chem..

[55] Kedashiro S., Sugiura A., Mizutani K., Takai Y. (2019). Nectin-4 cis-interacts with ErbB2 and its trastuzumab-resistant splice variants, enhancing their activation and DNA synthesis. Sci. Rep..

