# Supplementary material for: Nectin-4 as a Predictive Marker for Poor Prognosis of Endometrial Cancer with Mismatch Repair Impairment
Source: Cancers (Basel). 2023 May 22;15(10):2865. doi: 10.3390/cancers15102865 (PMC10216516; doi:10.3390/cancers15102865)
Supplement: Supplementary file 1 [file cancers-15-02865-s001.zip › cancers-2381552-supplementary.pdf]

Supplementary Materials

Nectin-4 as a predictive marker for poor prognosis of endometrial cancer with mismatch repair impairment

Ha Kyun Chang, Young Hoon Park, Jung-A Choi*, Jeong Won Kim, Jisup Kim, Hyo Sun Kim, Hae Nam Lee, Hanbyoul Cho, Joon-Yong Chung, and Jae-Hoon Kim*

Figure S1. Alteration frequency of ERBB2 and ERBB3 is associated with Nectin-4 expression in endometrial cancer. (**A**) Gene alteration frequency of ERBB proteins in TCGA PanCancer Atlas Studies was analyzed using the cBioPortal tool. (**B**) Correlation between mRNA expression of Nectin-4 and ERBBs in endometrial cancer tumors from TCGA PanCancer Atlas Studies using the cBioPortal tool. The expression value is presented as the Z-score fold-change RNA-seq expression (v2 RSEM). The Spearman's correlation rank for each comparison is shown, and significance is shown using a *p*-value.

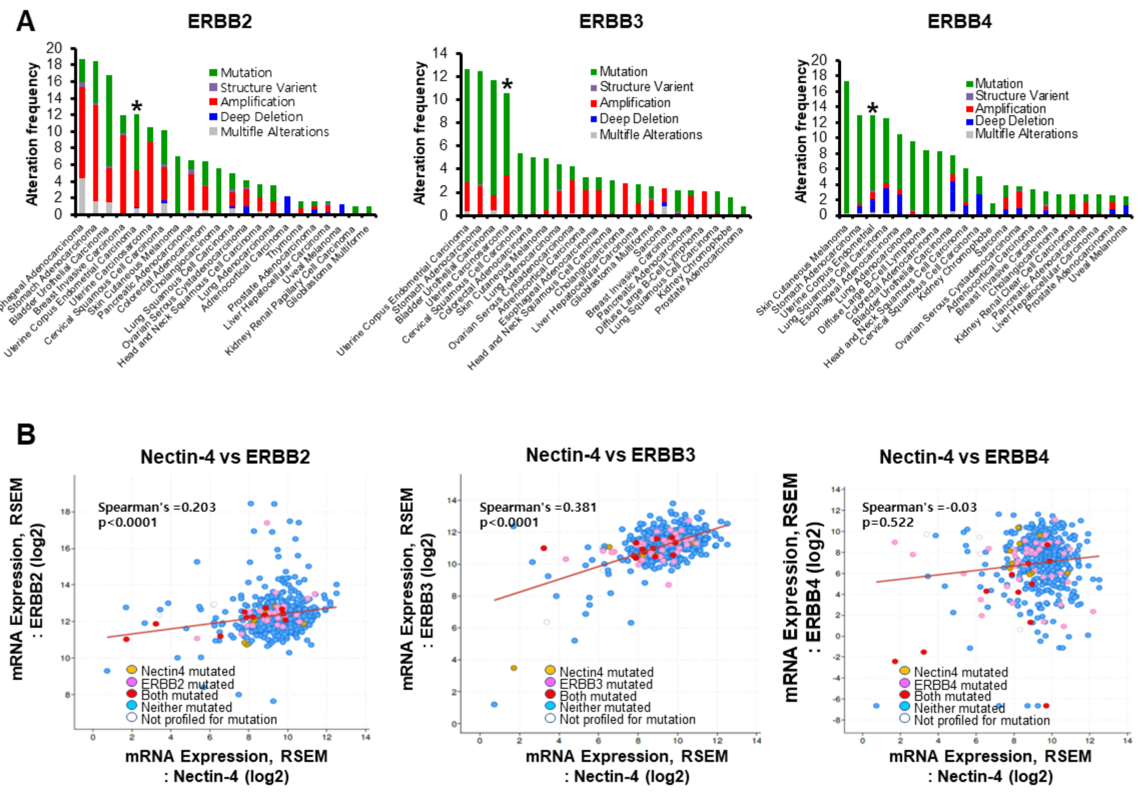

Table S1. Inclusion and exclusion criteria for the retrospective study.

Inclusion criteria	1. Patients diagnosed with endometrial cancer or benign tumor from January 2005 to November 2021<br>2. Patients ≥ 19 years old<br>3. Subjects who have agreed to provide tissues to the human material bank during surgery and biopsy to be performed for the purpose of treatment and diagnosis
Exclusion criteria	1. History of infections or other serious medical problems that impair the patient's function and make protocol compliance difficult.<br>2. Patients judged by the investigator that they should not participate in the clinical trial because they will not be able to comply with the clinical trial procedures and requirements

Table S2. Ratio of groups of Nectin-4^{High} and Nectin-4^{Low} in CD8-positive patients with MSH-deficient endometrial cancer.

**Groups**		**% of CD8-positive patients**	
		Ratio (*n*)	Total
MSH2-deficient	Nectin-4^{High}	16.7% (1)	6
	Nectin-4^{Low}	83.3%(5)	
MSH2-proficient	Nectin-4^{High}	78.9% (30)	38
	Nectin-4^{Low}	21.1% (8)	
MSH6-deficient	Nectin-4^{High}	25% (2)	8
	Nectin-4^{Low}	75.0% (6)	
MSH6-proficient	Nectin-4^{High}	80.6% (29)	36
	Nectin-4^{Low}	19.4% (7)	
